# Nasal Polypus—A Clinical Case

**Published:** 1899-03

**Authors:** F. S. Davis

**Affiliations:** Quincy, Mass.


### NASAL POLYPUS—A CLINICAL CASE.

By F. S. Davis, M. D., Quincy, Mass.

October 28th, 1897.—Mr. T. L. W., a strong healthy Englishman æt. 47, dark complexioned, dark eyes and hair, large frame, good weight—183 pounds—and ruddy face, came to my office with the following history:

Always enjoyed good health; two years ago worked at stone cutting for several years; now works on watch repairing. At age of eleven months had small-pox; was purposely exposed by his father. Two years ago began to have sneezing spells, with obstruction of nose—coming suddenly—could hardly breathe. Had his nose examined by specialist, who diagnosed polypi, which he proceeded to remove. He was told he would have to come again for treatment, as the growths would return. He was treated sixteen times in six months, getting relief for a time only, the doctor telling him there was no cure for them except to keep cutting until they got tired of growing.

He was telling me of his experience, and I informed him that the trouble could be cured with medicine. As he was at this time trying to get his courage up for another treatment by the specialist he was glad, he said, to place his case in my hands.

On examination nose presented a swollen appearance which destroyed the natural shape, and internally it was red and swollen, making it difficult to examine the growths, but they could be seen. Said he had pneumonia fifteen years ago; had been well since except at times could not sleep; had taken every kind of sleeping powder and various catarrh cures, but with no benefit.

For two or three years has had diarrhœa spells, coming on suddenly, day or night; painless mostly, except little gripe at first and slight trouble with piles; slight rheumatic pains in left knee and right shoulder only occasionally. Subject to catarrhal trouble in posterior nares—dropping of mucus in throat. His uncle had polypi at same age. Fine capillary vessels very marked over his face. Had pedunculated warts removed from back. When a boy had warts on hands. Now discharges clear water; will sneeze so constantly he cannot work; itching on back of hands but never finds any reason for it. His father contracted itch before marriage. ℞—Sulphur, CM., a powder, night and morning for a few days, then Placebo.

November 4th.—Reported improvement; spells of sneezing much less severe, and less frequent; breathing easier; to-day, had a little diarrhœa—yellow stools. Placebo.

November 27th.—Better, until to-day, when a sudden sneezing spell came on at 8 P. M., with tingling in nose and throat; water discharge from nose drops if head is held down. Last night, on blowing the nose, had a firm mass come away, that had a hard feel to touch, and felt fleshy. Did not see it to examine it. Nose felt free after this except the watery discharge continued; nose is free now. Tells me he found a small pedunculated wart on his back, and that he once had a wart removed from his little finger, years ago. Feet are very dry. Placebo.

December 12th.—No change in the head symptoms; feels cross and aggressive; can't stand any disturbance. He says this is very new for him; constipation. Nux-v. 200.

December 26th.—Has been so troubled with nose being obstructed he could not sleep; sneezing almost continuously; comes on as soon as he gets out of bed, better as soon as he lies down. Exhibits his arms covered with wen-like tumors, some large, others small; also on back and thighs. They have been there a long time—fifteen years.

I gave Placebo, requesting a report in three days, if not better.

December 29th.—No better; sneezing and other symptoms continue. As I had been looking up a remedy, by the help of Dr. O. M. Drake's very complete repertory of polypoid growths, and as my patient seemed to get an aggravation from damp weather, also the nose being sore, which was much increased by touching it, I gave Marum-verum, 1M—a few doses, followed by Placebo.

January 19th.—Reports not much change in head and nose, but calls my attention to a new line of symptoms. The angles of the ribs are so sensitive to pressure of the bed that he cannot lie down in bed; must relieve these parts of pressure; has had more or less of this for the past fifteen years. Heels will be sensitive to pressure of bed; also the knees if they press on each other—old symptoms. Ears will not endure pressure of pillow—old symptom. Heels are tender to pressure on standing, also soles of feet; frontal headache mornings on waking—a dull pain, with slight nausea, with disgust at food. I gave Medorrhinum, 1M.

January 27th.—Head quite clear; very little sneezing; sleep some better. Had diarrhœa at 4 P. M. and at 8 P. M yesterday—stool yellow with much flatus, and some griping pain, relieved after stool; good sleep last night—free from headache to-day. Ribs still feel sore lying in bed; very sensitive to noise at any time. Placebo.

February 23d.—Has had no headache; better appetite than for some time; sleep much better; stool natural; ribs still trouble; hips seem to get sore in bed; less rheumatic pains; head clear. Placebo.

March 9th.—Reports better in every way; has had no trouble with nose; it begins to look natural in shape; breathing is perfectly free; no headache. Placebo.

April 10th.—Only troubled by the soreness of ribs at night; no soreness of the feet; nose free; no watery discharge. It feels natural; nothing to complain of. Placebo.

April 17th.—In walking, has noticed a tendency for the toes to flex; will not notice it until a painful tiredness comes on; a tendency for the jaws to be firmly closed; will not notice it until they begin to ache from the pressure. Wen-like tumors seem less firm, and it does not hurt to press on them as it used to do; nose looks in natural shape; feet now perspire and feel natural. Placebo.

May 1st.—Itching of ear quite marked about the edges; toes and ribs still troublesome; some nights is so nervous and restless can't sleep; jaws have been natural; had another firm mass expelled from the nose—looked like putty. Placebo.

May 11th.—Improvement. Placebo.

May 26th.—Has two attacks of diarrhœa, coming on in the afternoon—one each week after eating rhubarb. He says rhubarb always causes diarrhœa. I gave one dose of Rheum. 1M.

June 2d.—Reports has had no more diarrhœa; nose feels good. Placebo.

June 19th.—Has had more loose stools, yellow color, coming on 3 to 4 o'clock P. M., and again at 10 P. M., on June 5th, and again on the 10th of June, and also on the 11th of June; June 13th had slight pain in bowels, but no stool; bowels natural since; has had, for days past, itching over middle of chest and at border of ribs, and on the arms near elbow joints; otherwise feels well except the sides trouble him in bed, and he has had some dull pain, at times, in the chest near border of ribs. Placebo.

This case is still under observation. The nose gives no trouble but on the contrary is freer than for past three or four years, and of very much better shape, and his general health is improving.

DISCUSSION.

Dr. Kennedy—The symptom mentioned by Dr. Davis in his paper, viz.: "Inability to keep the feet still," is very suggestive of another remedy—Zinc. I wish to ask the doctor if he was led to the remedy by that symptom.

Dr. Davis—That symptom being characteristic of Zinc, I consulted the guiding symptoms under Zinc, and there found a close correspondence of not only the verified symptons, but also those marked as less characteristic, which left no doubt in my mind that this was the simillimum, or at least a close similar, and it is proving curative.

